# Research on the optimal selection method for railway line routes in complex and challenging mountainous areas taking into account ecological protection

**DOI:** 10.1371/journal.pone.0341632

**Published:** 2026-02-20

**Authors:** Feng Han, Yunxiang Luo

**Affiliations:** School of Civil Engineering, Lanzhou Jiaotong University, Lanzhou, China; Novum Research and Innovation Group, LEBANON

## Abstract

This study addresses the coordinated optimization of ecological conservation and engineering efficiency in railway route planning through challenging mountainous terrain, proposing a selection methodology that prioritizes environmental protection. A comprehensive evaluation system with 20 indicators was developed across technique, economy, environment, and social dimensions. Key considerations include impacts on ecologically sensitive zones, engineering risks, and low-carbon development objectives. The weighting system integrates expert knowledge (G2 method) and data patterns (CRITIC method), optimized through relative entropy to balance subjective and objective information, enhancing weighting reliability. A decision-making model was established using trapezoidal fuzzy numbers for qualitative indicators and multi-attribute utility theory. Projection values and comprehensive relative closeness calculations enable quantitative scheme evaluation. Applied to a high-speed railway crossing protected areas, the model validated Scheme II (full-tunnel passage through sensitive zones) as optimal (0.5202 relative closeness), demonstrating cost efficiency, minimal environmental impact, and enhanced safety – consistent with practical engineering outcomes. The methodology provides a scientifically grounded decision-making framework balancing ecological preservation and engineering viability, supporting green transportation transformation under carbon neutrality goals. Combination weighting.

## 1. Introduction

Global climate change mitigation efforts and China’s carbon peaking/neutrality strategy have intensified focus on transportation – the world’s third-largest carbon emitter (16% of global emissions) – driving urgent needs for sustainable transformation [1]. By 2024, China’s railway network expanded to 162,000 km (48,000 km high-speed rail), yet this rapid growth has exacerbated ecological impacts from conventional planning approaches [2]. Recent data reveals critical gaps: 38.7% of new railways traverse ecologically sensitive zones, habitat fragmentation increases 12.4% annually, and construction emits 0.87 tons CO₂/km [3]. This underscores the imperative to integrate ecological considerations into route planning. Aligned with China’s Transport Power Strategy, systematic actions are required to implement low-carbon solutions, accelerate emission reduction targets, and advance sustainable railway development [4]. Conventional mountainous route planning faces technical and ecological challenges: rugged terrain, geological risks, and outdated environmental assessments. A paradigm shift toward proactive ecological protection in design processes is essential [5]. This context establishes ecologically conscious railway planning in challenging terrains as a critical research frontier for sustainable infrastructure development.

Current railway route selection research demonstrates progress in weighting methodologies. Scholars like Yang Wenxin et al. [6] employed the Analytic Hierarchy Process (AHP), while Liu Runkai et al. [7] combined AHP with entropy weighting. These structured approaches effectively integrate expert insights and quantify environmental impacts, gaining broad acceptance in transportation studies. However, their reliance on subjective weight determination introduces significant human bias.In decision modeling, Gao Yuxiang et al. [8] utilized cloud models and enhanced TOPSIS techniques, ranking alternatives via closeness coefficients to ideal solutions. While effective, this approach shows sensitivity to data normalization and outlier influence. Comparatively, Liang Dong’s [9] osculating value method evaluates both optimal and suboptimal benchmarks, demonstrating strength in multi-criteria analysis but remaining highly weight-dependent – a critical limitation as biased weights may skew outcomes.

To address these limitations, this study employs a dual-weighting approach: The G2 method for subjective weight determination and the CRITIC method for objective weighting. These are integrated through relative entropy optimization, effectively balancing expert judgment and data-driven insights while mitigating individual biases. A utility-based decision model enhances objectivity by systematically evaluating alternatives against national economic priorities. This framework adapts to policy-driven risk preferences (risk-averse or risk-preferring strategies), offering robust technical support for railway route selection in ecologically sensitive mountainous regions.

## 2. Study area

**Fig 1:**
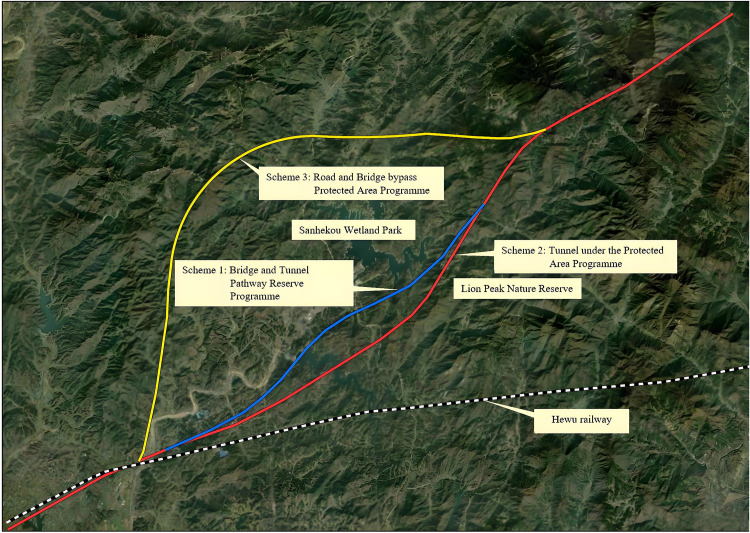
Schematic diagram of the line scheme.

A case study was conducted to validate the proposed model’s applicability in the Dabie Mountains, a geologically complex region with widespread environmentally sensitive zones and rockfall hazards. To balance ecological preservation and operational safety, three alternative routes (Fig 1) were analyzed through technical feasibility assessments. The schemes are summarized as follows:

**Scheme I**: Originating from the Dabie Mountain Tunnel portal in Anhui Province, the route crosses the Hubei-Anhui provincial border westward, traverses the experimental zone of Shizifeng Nature Reserve, Sanhekou Provincial Wetland Park, and the secondary water protection area of Sanhekou Reservoir. It then passes downstream of the Bilühe Reservoir dam before crossing Yanjia River and connecting to the existing Hefei-Wuhan Railway. **Scheme II**: Following the same origin, this route employs a full-tunnel configuration (Yinkongshan Tunnel: 5.42 km) beneath the Shizifeng Nature Reserve’s experimental zone and Sanhekou Wetland Park’s utilization area. It crosses the mid-section of Bilühe Reservoir and Yanjia River before linking to the Hefei-Wuhan Railway. **Scheme III**: Diverging northwest at the Henan provincial border, this route bypasses critical ecological zones via bridges and elevated structures along the northern periphery of Sanhekou Wetland Park, later aligning with Scheme I’s southwestern trajectory. Detailed engineering parameters are provided in Table 1.

**Table 1:** Specific project information.

Project information	I	II	III
Line length (km)	34.802	35.023	48.562
Bridge-to-Tunnel ratio (%)	91.44	97.35	90.78
Post-operation and maintenance costs	Larger	Smaller	Large
Total length of mega-bridges and large and medium-sized bridges (km)	10.163	7.744	18.341
Length of Grown-up Tunnel (km)	14.793	20.221	17.873
Length of Railway bed (km)	2.98	0.926	7.86
Quantity of earthwork and stonework (m^3^)	250870	34100	84700
Volume of land requisitioned (m^2^)	432600	324666.67	577266.67
Demolition of buildings (m^2^)	25430000	24000	26480000
Project Investment Estimates (yuan)	4625985000	4598156000	4957134000
Impacts on ecosystems in nature reserves (km)	10790.798	8841.41	9415.842
Impacts on rare and endangered wildlife and plants	Larger	Small	Smaller
Impact of poor geology on engineering	Larger	Smaller	Generic
Impacts on landscape resources	Large	Larger	Larger
Impacts on water systems	Larger	Small	Large
Ability to attract passenger and cargo flows	Better	Better	Poor
Promoting economic development	Good	Better	Poor
Implications for urban planning layout	Better	Good	Generic
Impact on integrated resource development	Better	Good	Poor

## 3. Methodology

### 3.1. Construction of comprehensive evaluation indicator system for complex and dangerous mountain railway route programmes

#### 3.1.1. Analysis of factors affecting the design of railway alignment in complex and dangerous mountainous areas.

Complex and hazardous mountainous regions are characterized by rugged terrain, harsh environments, and limited accessibility. These areas, often situated in ecologically sensitive zones with fragile ecosystems, are shaped by geological processes such as tectonic movements and rock layer transformations [10]. Railways traversing these regions face significant challenges to operational safety and passenger comfort. Railway construction in such areas poses ecological risks, particularly within protected zones, including habitat fragmentation, biodiversity loss, soil erosion, noise/air pollution, invasive species introduction, light pollution, and wildlife-vehicle collisions [11]. To address these challenges, eco-conscious route planning for high-speed railways should prioritize minimizing protected-area crossings and leveraging natural topography to reduce environmental impacts.

The geological complexity of hazardous mountainous regions presents unique construction challenges compared to standard mountain terrain. Railway design in these areas must account for frequent landslide risks during extreme weather events and inherent construction difficulties. Existing case studies demonstrate two effective strategies for minimizing environmental impact: direct tunneling through sensitive zones or routing with wide-radius curves to bypass critical areas [12]. Protective infrastructure along mountain routes is essential for operational safety during geological hazards. Where such measures prove impractical, selective terrain modification may be implemented to ensure safe rail operations.

Hazardous mountainous regions feature steep peaks and cliffs as dominant landforms. However, short-term planning, superficial assessments, and profit-driven priorities in high-speed rail construction have exacerbated ecological damage in these fragile ecosystems, often causing irreversible harm due to insufficient understanding of environmental vulnerabilities [13]. Route planning must prioritize ecological preservation strategies to minimize environmental degradation. Passenger needs remain central to high-speed rail design, requiring careful optimization of route configurations to ensure travel comfort and service quality. A holistic evaluation during preliminary planning phases is essential to enhance operational efficiency while balancing ecological protection and regional development objectives.

#### 3.1.2. Construction of the indicator system.

**Fig 2:**
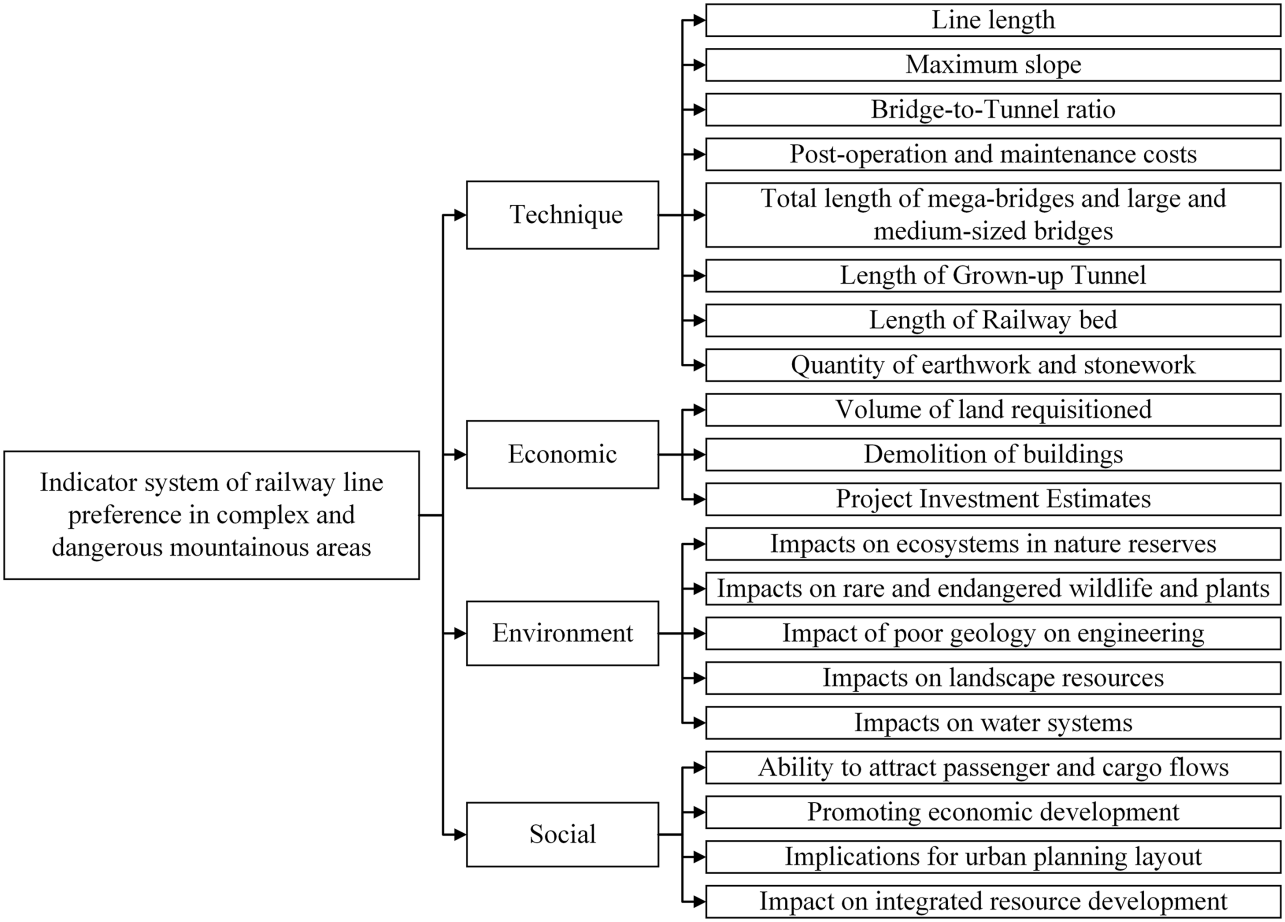
Indicator system of railway line preference in complex and dangerous mountainous areas.

Route planning outcomes vary across railway projects due to differing contextual priorities and environmental constraints. A robust evaluation framework must underpin decision-making processes to ensure methodological rigor and outcome reliability [14]. This study integrates the unique geographical challenges of hazardous mountainous terrain with high-speed rail alignment principles, establishing a four-dimensional evaluation framework (technical, economic, environmental, social) for route optimization, as detailed in Fig 2. Environmental impact on protected ecosystems is quantified through infrastructure penetration distance within conservation zones.

### 3.2. Multi-attribute utility theory decision modelling

#### 3.2.1. Trapezoidal fuzzy number concept.

The proposed evaluation framework integrates both qualitative and quantitative indicators. While quantitative metrics enable direct numerical comparison of alternatives, qualitative criteria lack standardized quantification methods, introducing subjectivity and uncertainty during decision-making. To address this limitation, trapezoidal fuzzy numbers [15] are employed to systematically convert ambiguous linguistic assessments into quantifiable values. This is achieved through membership functions and semantic mapping rules. This approach resolves the persistent challenge of qualitative indicator quantification in traditional systems, enabling standardized processing of heterogeneous criteria and enhancing the objectivity and comparability of multi-alternative evaluations.

(1) Definition of trapezoidal fuzzy numbers

Consider a universe of discourse *U*. Let *A* denote a set of real numbers within *U*, where $r_{\text{1}},r_{\text{2}},r_{\text{3}},r_{\text{4}}$  ∈ A satisfies condition $0\leq r_{\text{1}}\leq r_{\text{2}}\leq r_{\text{3}}\leq r_{\text{4}}$. A trapezoidal fuzzy number $M=\left(r_{\text{1}},r_{\text{2}},r_{\text{3}},r_{\text{4}}\right)$ is defined by its membership function $M_{A}\left(x\right)$, which maps elements of *A* to their corresponding membership degrees.

(2) Distance between trapezoidal fuzzy numbers

Let $M_{\text{1}}=\left[r_{\text{1}},q_{\text{1}},s_{\text{1}},t_{\text{1}}\right]$, $M_{\text{2}}=\left[r_{\text{2}},q_{\text{2}},s_{\text{2}},t_{\text{2}}\right]$ be two trapezoidal fuzzy numbers, then define


$$d\left(M_{\text{1}},M_{\text{2}}\right)=\frac{\left|r_{\text{1}}-r_{\text{2}}\right|+\left|q_{\text{1}}-q_{\text{2}}\right|+\left|s_{\text{1}}-s_{\text{2}}\right|+\left|t_{\text{1}}-t_{\text{2}}\right|}{4n}$$ (1)

is the distance between $M_{\text{1}},M_{\text{2}}$. where *n* denotes the number of linguistic variables contained in the set *M* of linguistic variables. When the value of $d\left(M_{\text{1}},M_{\text{2}}\right)$ is greater, the distance between $M_{\text{1}}$ and $M_{\text{2}}$ is greater.

**Fig 3:**
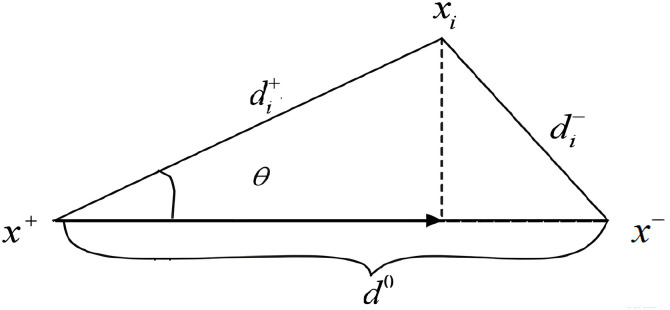
Positive and negative bull’s-eye and integrated bull’s-eye distance for arbitrary scheme.

Since the distance from any option $X_{i}$ to the optimal option satisfies $d_{i}^{+}\leq d^{\text{0}}$ and the distance to the worst option satisfies $d_{i}^{-}\leq d^{\text{0}}$, any option point is in the body enclosed by surface $AZ^{+}A^{\text{'}}$ and surface $AZ^{-}A^{\text{'}}$, then the $d_{i}^{+},d_{i}^{-},d^{\text{0}}$ space triple of any alternative option point falls on the same straight line or encloses a triangle, as shown in Fig 3:

From Fig 3, it can be seen that the optimal programme point $X_{i}$, the closer it is to the positive bullseye point $x^{+}=\left(x_{\text{1}}^{+},x_{\text{2}}^{+},\ldots ,x_{n}^{+}\right)$, and the further it is to the negative bullseye point $x^{-}=\left(x_{\text{1}}^{-},x_{\text{2}}^{-},\ldots ,x_{n}^{-}\right)$. However, due to the discrepancy between the alternative programme points and the ideal programme, the three points of the alternative programme points $d_{i}^{+},d_{i}^{-},d^{\text{0}}$ are usually surrounded by triangles, which is not conducive to judging the true degree of its superiority or inferiority. Therefore, the projection of the positive bull’s-eye distance on the line connecting the positive and negative bull’s-eye is defined as the integrated bull’s-eye distance $d^{*}$ to visually characterize the distance between the alternative and the positive and negative bull’s-eye, which can be obtained due to the cosine theorem, and the specific calculation formula is:


$$(d_{i}^{+}{)}^{\text{2}}+({d\;}^{\text{0}}{)}^{\text{2}}-2d_{i}^{+}d^{\text{0}}\;cos\;\theta =(\varepsilon_{i}^{-}{)}^{\text{2}}$$ (2)

$$cos\;\theta =\frac{(d_{i}^{+}{)}^{\text{2}}+(d^{\text{0}}{)}^{\text{2}}-(d_{i}^{-}{)}^{\text{2}}}{2d_{i}^{+}d^{\text{0}}}$$ (3)

$$d^{*}=d_{i}^{+}cos\;\theta =\frac{{\left(d_{i}^{+}\right)}^{\text{2}}+{\left(d^{\text{0}}\right)}^{\text{2}}-{\left(d_{i}^{-}\right)}^{\text{2}}}{2d^{\text{0}}}$$ (4)

In order to make full use of the valid information implied by the alternatives, a distance superiority degree $\sigma_{i}$ was defined to measure the degree of superiority of each alternative based on the combined bull’s-eye distance of the points of the alternatives, which was calculated as follows:


$$\sigma_{i}=1/\left(1+d^{*}\right)\text{\textbackslash{}hspace\{1em}\}i=\left(1,2,\cdots ,m\right)$$ (5)

where the larger the $\sigma_{i}$, the better the benefits derived from the indicator.

#### 3.2.2. Weighting.

This study establishes a hybrid weighting system that integrates expert preferences with data-driven insights, effectively capturing the interplay between subjective judgments and objective patterns. The methodology combines the G2 method for subjective weighting and the CRITIC technique for objective weighting, unified through Kullback-Leibler divergence optimization. The G2 method employs pairwise comparisons by domain experts to construct precedence relationship matrices, systematically codifying subjective priorities through reference-based ordinal analysis [16]. The CRITIC method quantifies indicator relevance through standard deviations (contrast intensity) and inter-indicator correlations (conflict analysis), enabling statistically grounded weight allocation [17]. A Kullback-Leibler divergence model optimizes the weighting integration by minimizing discrepancies between subjective and objective distributions [18], implemented through the following computational procedure:

(1) Dimensional empowerment based on personal experience:

① The expert or decision maker gives the ratio *s*_*tn*_ of the degree of importance of the evaluation indicator *S*_*jt*_ to *S*_*jn*_ as follows:


$$s_{tn}≜a_{t},t=1,2,\ldots ,n$$ (6)

Included among these, rational assignment $a_{t}>0,a_{n}=1$.

② Calculate the weight $W_{jt}$ of the *j*_*t*_ evaluation indicator according to the rational assignment *a*_*t*_ with the following formula:


$$W_{jt}=a_{t}/\sum \begin{array}{c} @ln\\ i=1 \end{array}a_{i},t=1,2,\ldots ,n$$ (7)

(2) Dimension assignment based on actual data:

① Construct the decision matrix based on the actual data, and obtain the matrix *K* after standardization.

② Calculate the coefficient of variation fluctuation of indicators $\zeta_{j}$ based on the standard deviation.


$$\zeta_{j}=\frac{\sqrt{\frac{\text{1}}{s-1}\sum_{i=1}^{s}\left(k_{ij}-\overset{―}{k_{ij}}\right)}}{\overset{―}{k_{j}}},j=1,2,3,\cdots ,t$$ (8)

where *s* is the number of programmes, *t* is the number of decision-making indicators, *k*_*ij*_ is the value of the *j* indicator of the *i* programme in the standardized matrix *K*, and $\overset{―}{kj}$ is the mean value of the *j* indicator.

③ Construct the matrix based on the Pearson correlation coefficient between the indicators, and then calculate the conflict coefficient $\zeta_{j}$ of each indicator:


$$f_{mn}=\frac{\sum \begin{array}{c} @lb\\ i=1 \end{array}\;\;\;(k_{im}-\overset{―}{k_{m}})(k_{in}-\overset{―}{k_{n}})}{\sqrt{\sum \begin{array}{c} @lb\\ i=1 \end{array}\;\;\;(k_{im}-\overset{―}{k_{m}}{)}^{\text{2}}}\sqrt{\sum \begin{array}{c} @lb\\ i=1 \end{array}\;\;\;(k_{in}-\overset{―}{k_{n}}{)}^{\text{2}}}}$$ (9)

④ Determine the objective weights *W*_*j*_ of the indicators by calculating the amount of information *C*_*j*_ of the indicators:


$$W_{j}=\frac{C_{j}}{\sum \begin{array}{c} @lb\\ j=1 \end{array}C_{j}}$$ (10)

(3) In order to rationalize the weights more, this paper adopts the relative entropy of multi-attribute decision-making combination assignment, based on the closeness of the results of each assignment to determine its weighting coefficient in the combination of weights, where the closeness refers to the relative entropy of the weight vector, and the steps for the calculation of the combination assignment are as follows:

① Combine the subjective and objective weight values calculated by m subjective and objective weighting methods to form the indicator weight vector:


$$v_{j}=\left(v_{j1},v_{j2},\cdot \cdot \cdot ,v_{j\text{n}}\right)\text{\textbackslash{}hspace\{0.17em}\}\left(j=1,2,\cdots ,m\right)$$ (11)

② Establish the optimization model and apply Eq (12) to calculate the agglomeration weights of *n* indicators $d^{*}=\left(d_{\text{1}}^{*},d_{\text{2}}^{*},\cdots ,d_{n}^{*}\right)$;


$$d_{i}^{*}=\prod_{j=1}^{m}(v_{ji}{)}^{\frac{\text{1}}{m}}/\sum_{i=1}^{n}\prod_{j=1}^{m}(v_{ji}{)}^{\frac{\text{1}}{m}}\text{\textbackslash{}hspace\{0.17em}\}i=1,2,\cdots ,n$$ (12)

③ Apply Eq (13) to calculate the closeness of each assignment result to the set weights $h(v_{i},d*)$ (i=1,2,⋯,m);


$$h(v_{i},d*)=\sum_{l=1}^{n}v_{il}\;ln\frac{v_{il}}{d_{l}^{*}}$$ (13)

④ Determine the credibility of each assignment result by combining Eq (14) on the basis that the closeness is known $\alpha_{i}$. From the physical significance of credibility, it can be seen that: the greater the closeness of a certain assignment result to the set weights, the greater the role it proves to play in the combination of assignments.


$$\alpha_{i}=h(v_{i},d^{*})/\sum_{i=1}^{m}h(v_{i},d^{*})\text{\textbackslash{}hspace\{0.17em}\}i=1,2,\cdots ,m$$ (14)

⑤ Calculate the combined weight value $w_{jc}$ of the evaluation indicators using Eq (15).


$$w_{jc}=\sum_{k=1}^{m}\alpha_{k}\;v_{kj}\text{\textbackslash{}hspace\{0.17em}\}\;j=1,2,\cdots ,n$$ (15)

#### 3.2.3. A decision-making model based on multi-attribute utility theory.

Railway route optimization constitutes a complex multi-attribute decision-making process requiring multidimensional analysis. To address limitations in conventional models – including inconsistent metric scaling, insufficient quantitative assessments, and underutilized alternative scheme data – this study employs utility theory to develop a decision framework. The methodology evaluates alternatives through comprehensive relative closeness, calculated via projection distances to positive/negative ideal solutions.

Modern utility theory originated from the groundbreaking work of John von Neumann and Oskar Morgenstern, who established the expected utility framework for uncertainty-driven decision-making. Their axiomatic approach formally demonstrated the existence of utility functions while defining operational rules and logical structures, forming the foundation of contemporary decision theory [19,20]. The relative closeness calculation procedure comprises the following steps:

The set of railway line options $\left\{A_{\text{1}},A_{\text{2}},\ldots ,A_{m}\right\}$ to be compared, the set of decision indicators $\left\{C_{\text{1}},C_{\text{2}},\ldots ,C_{n}\right\}$ and the weight vector $\left(\omega_{\text{1}},\omega_{\text{2}},\ldots ,\omega_{n}\right)$ for each indicator are first determined.

(1) Indicator normalization

When the indicator takes the value of the exact number $a_{ij}$, it is transformed into $s_{ij}$ according to the utility function.


$$s_{ij}=\frac{a_{ij}-(d_{min}^{\;j}-d_{\text{1}}^{\;j})}{\left(d_{max}^{\;j}+d_{\text{2}}^{\;j})-(d_{min}^{\;j}-d_{\text{1}}^{\;j}\right)}=\frac{a_{ij}-x_{min}^{\;j}}{x_{max}^{\;j}-x_{min}^{\;j}}$$ (16)

where $d^{\;j}$ is defined as the domain of taking values $d_{\text{1}}^{\;j}$ and $d_{\text{2}}^{\;j}$ are two suitable positive numbers.

(2) Projections of the scheme to positive and negative ideal solutions

For the cost-based indicators, smaller values are defined as positive ideal scenario $v_{j}^{+}=\left(v_{\text{1}}^{+},v_{\text{2}}^{+},\ldots ,v_{n}^{+}\right)$, and larger values are defined as negative ideal scenario $v_{j}^{-}=\left(v_{\text{1}}^{-},v_{\text{2}}^{-},\ldots ,v_{n}^{-}\right)$. For the benefit-based indicators, larger values are defined as positive ideal scenarios, and smaller values are defined as negative ideal scenarios, obtaining the projected values of each scenario in terms of positive and negative ideal scenarios $Pr\;j_{A^{+}}\left(X_{i}\right)$ and $Pr\;j_{A^{-}}\left(X_{i}\right)$.


$$Pr\;j_{A^{+}}\left(X_{i}\right)=\frac{\sum_{j=1}^{n}\omega_{i}s_{ij}v_{j}^{+}}{\sqrt{\sum_{j=1}^{n}(v_{j}^{+}{)}^{\text{2}}}}$$ (17)

$$Pr\;j_{A^{-}}\left(X_{i}\right)=\frac{\sum_{j=1}^{n}\omega_{i}s_{ij}v_{j}^{-}}{\sqrt{\sum_{j=1}^{n}(v_{j}^{-}{)}^{\text{2}}}}$$ (18)

(3) Solve for the integrated relative closeness *P*_*i*_.


$$P_{i}=\frac{Pr\;j_{A^{+}}\left(X_{i}\right)}{Pr\;j_{A^{+}}\left(X_{i}\right)+Pr\;j_{A^{-}}\left(X_{i}\right)},i=1,2,\ldots ,n$$ (19)

Finally, the schemes are ranked according to the size of the *P*_*i*_ value, where a larger *P*_*i*_ value indicates that the scheme is closer to the ideal scheme.

## 4. Results

### 4.1. Weight determination

A well-optimized route alignment not only mitigates construction risks and costs but also enhances operational safety while reducing long-term maintenance expenditures. The three proposed schemes exhibit distinct characteristics with significant variations in performance metrics, necessitating a comprehensive multi-criteria analysis to objectively identify the optimal solution.

Qualitative indicators were systematically converted into trapezoidal fuzzy numbers using the transformation framework presented in Table 2, enabling consistent integration of expert judgments with quantitative data.

**Table 2:** Trapezoidal fuzzy number transformation relationship.

Cost-based indicators	Benefit-based indicators	trapezoidal fuzzy number
Very large	Very poor	(0,0,0,0.091)
Larger	Poorer	(0,0.091,0.182,0.273)
Large	Poor	(0.182,0.273,0.364,0.455)
llGeneric	Generic	(0.364,0.455,0.545,0.636)
Small	Good	(0.545,0.636,0.727,0.818)
Smaller	Better	(0.727,0.818,0.909,1)
Very small	Very good	(0.909,1,1,1)

According to Eqs (6)–(10) based on the subjective experience dimension as well as the objective data dimension are assigned to obtain the subjective weight $\omega_{\text{1}}$ and objective weight $\omega_{\text{2}}$, respectively.


$$\begin{array}{c} \omega_{\text{1}}=\left\{ \begin{array}{cccccccccc} @cccccccccc@0.0457 & 0.0423 & 0.0305 & 0.0372 & 0.0338 & 0.0271 & 0.0169 & 0.0203 & 0.0237 & 0.0508 \end{array}\right.\mathrm{\backslash hfill}\\ \;\;\text{\textbackslash{}hspace\{0.33em}\;\;\}\begin{array}{ccccccccc} 0.0931 & 0.0880 & 0.0728 & 0.0761 & 0.0846 & 0.0711 & 0.0660 & 0.0626 & 0.0575 \end{array}\;\;\;\}\mathrm{\backslash hfill} \end{array}$$

$$\begin{array}{c} \omega_{\text{2}}=\left\{ \begin{array}{cccccccccc} @cccccccccc@0.0419 & 0.0737 & 0.0729 & 0.0402 & 0.0516 & 0.0403 & 0.0740 & 0.0413 & 0.0587 & 0.0413 \end{array}\right.\mathrm{\backslash hfill}\\ \;\;\text{\textbackslash{}hspace\{0.33em}\;\;\}\begin{array}{ccccccccc} 0.0713 & 0.0515 & 0.0548 & 0.0494 & 0.0732 & 0.0423 & 0.0365 & 0.0439 & 0.0413 \end{array}\;\;\;\}\mathrm{\backslash hfill} \end{array}$$

The portfolio assignment $\omega^{*}$ is obtained by using the relative entropy method based on Eqs (11)–(15).


$$\begin{array}{c} \omega^{*}=\left\{ \begin{array}{cccccccccc} @cccccccccc@0.0434 & 0.0610 & 0.0557 & 0.0390 & 0.0444 & 0.0349 & 0.0508 & 0.0328 & 0.0445 & 0.0451 \end{array}\right.\mathrm{\backslash hfill}\\ \;\;\text{\textbackslash{}hspace\{0.33em}\;\;\}\begin{array}{ccccccccc} 0.0801 & 0.0663 & 0.0621 & 0.0603 & 0.0778 & 0.0540 & 0.0484 & 0.0515 & 0.0479 \end{array}\;\;\;\}\mathrm{\backslash hfill} \end{array}$$

**Fig 4:**
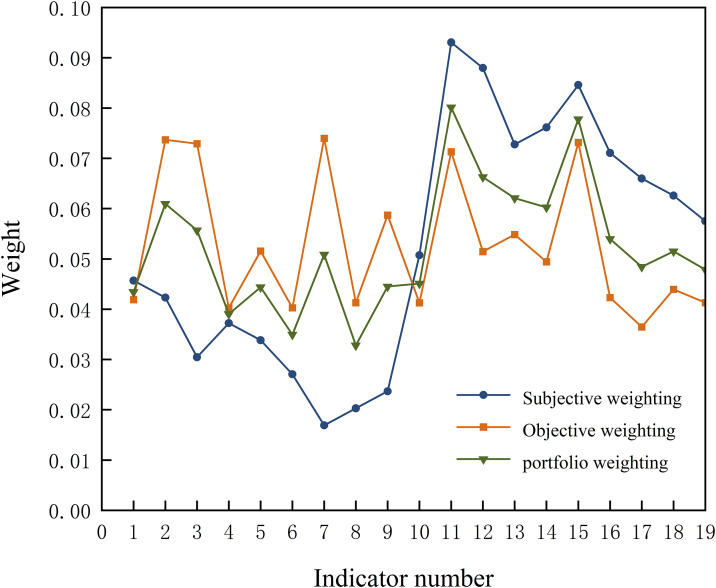
Changes in indicator weightings.

As shown in Fig 4, discrepancies exist between the CRITIC and G2 weighting methods due to minimal variations in indicator values across the three schemes. The CRITIC method assigns disproportionately low weights under these conditions, potentially diminishing its analytical effectiveness. To address this limitation and enhance alignment with practical engineering requirements, relative entropy optimization was applied to recalibrate the weighting system. The optimized composite weights demonstrate balanced integration of empirical and objective data-driven insights, yielding scientifically sound and reliable outcomes.

### 4.2. Scheme determination

According to Eqs (1)–(5)^‌‌^ the fuzzy numbers are transformed into quantifiable exact numbers by the method of cosine theorem to obtain the decision matrix A_1_.


$$\begin{array}{c} A_{\text{1}}\text{=}[\begin{array}{ccccccccccc} @lllllllllll34.802 & 91.44 & 0.603 & 10.163 & 14.793 & 2.98 & 25.087 & 648.9 & 2543 & 462598.5 & 10790.798\\ 35.023 & 97.35 & 0.772 & 7.744 & 20.221 & 0.926 & 3.41 & 487 & 2.4 & 459815.6 & 8841.41\\ 48.562 & 90.78 & 0.543 & 18.341 & 17.873 & 7.86 & 8.47 & 865.9 & 2648 & 495713.4 & 9415.842 \end{array}\\ \text{\textbackslash{}hspace\{0.33em}\;\;\;\;\;\;\;\}\begin{array}{cccccccc} @llllllll0.603 & 0.603 & 0.543 & 0.603 & 0.772 & 0.898 & 0.772 & 0.772\\ 0.898 & 0.772 & 0.603 & 0.898 & 0.772 & 0.772 & 0.898 & 0.898\\ 0.772 & 0.677 & 0.603 & 0.543 & 0.603 & 0.603 & 0.677 & 0.603 \end{array}\;\;] \end{array}$$

The utility processing of the decision matrix A_1_ according to Eq (16)^‌‌^ yields the utility matrix A_2_.


$$\begin{array}{c} A_{\text{2}}\text{=}[\;\begin{array}{ccccccccccc} @lllllllllll0.0072 & 0.1123 & 0.3725 & 0.2333 & 0.0178 & 0.3019 & 0.9954 & 0.4273 & 0.9603 & 0.0775 & 0.9999\\ 0.0230 & 0.9852 & 0.7666 & 0.0093 & 0.9822 & 0.0140 & 0.0046 & 0.0003 & 0.0000 & 0.0000 & 0.0001\\ 0.9928 & 0.0148 & 0.2334 & 0.9907 & 0.5650 & 0.9860 & 0.2359 & 0.9997 & 1.0000 & 1.0000 & 0.2947 \end{array}\\ \text{\textbackslash{}hspace\{0.33em}\;\;\;\;\;\;\;\}\begin{array}{cccccccc} @llllllll0.2020 & 0.2711 & 0.3852 & 0.2878 & 0.7289 & 0.7980 & 0.4628 & 0.5432\\ 0.7980 & 0.7289 & 0.6148 & 0.8197 & 0.7289 & 0.5432 & 0.7624 & 0.7980\\ 0.5432 & 0.4719 & 0.6148 & 0.1803 & 0.2711 & 0.2020 & 0.2376 & 0.2020 \end{array}] \end{array}$$

The utility matrix A_2_ is then assigned to obtain the positive ideal scenario $v_{j}^{+}$ and the negative ideal scenario $v_{j}^{-}$.


$$\begin{array}{c} v_{j}^{+}=\{0.0003\text{\textbackslash{}hspace\{1em}\}0.0009\text{\textbackslash{}hspace\{1em}\}0.0130\text{\textbackslash{}hspace\{1em}\}0.0004\text{\textbackslash{}hspace\{1em}\}0.0008\text{\textbackslash{}hspace\{1em}\}0.0005\text{\textbackslash{}hspace\{1em}\}0.0002\text{\textbackslash{}hspace\{1em}\}0.0000\text{\textbackslash{}hspace\{1em}\}0.0000\text{\textbackslash{}hspace\{1em}\}0.0000\\ \;\;\;\;\;\;0.0000\text{\textbackslash{}hspace\{1em}\}0.0134\text{\textbackslash{}hspace\{1em}\}0.0168\text{\textbackslash{}hspace\{1em}\}0.0232\text{\textbackslash{}hspace\{1em}\}0.0140\text{\textbackslash{}hspace\{1em}\}0.0393\text{\textbackslash{}hspace\{1em}\}0.0386\text{\textbackslash{}hspace\{1em}\}0.0393\text{\textbackslash{}hspace\{1em}\}0.0382\} \end{array}$$

$$\begin{array}{c} v_{j}^{-}=\{0.0431\text{\textbackslash{}hspace\{1em}\}0.0601\text{\textbackslash{}hspace\{1em}\}0.0427\text{\textbackslash{}hspace\{1em}\}0.0387\text{\textbackslash{}hspace\{1em}\}0.0436\text{\textbackslash{}hspace\{1em}\}0.0344\text{\textbackslash{}hspace\{1em}\}0.0506\text{\textbackslash{}hspace\{1em}\}0.0328\text{\textbackslash{}hspace\{1em}\}0.0445\text{\textbackslash{}hspace\{1em}\}0.0451\\ \;\;0.0801\text{\textbackslash{}hspace\{1em}\}0.0529\text{\textbackslash{}hspace\{1em}\}0.0453\text{\textbackslash{}hspace\{1em}\}0.0370\text{\textbackslash{}hspace\{1em}\}0.0638\text{\textbackslash{}hspace\{1em}\}0.0146\text{\textbackslash{}hspace\{1em}\}0.0098\text{\textbackslash{}hspace\{1em}\}0.0122\text{\textbackslash{}hspace\{1em}\}0.0097\} \end{array}$$

## 5. Validation and discussion

The projections and combined relative closeness of Scheme I, II and III on the positive and negative ideal scenarios are calculated according to Eqs (17)–(19)^‌‌^, respectively, and the results are shown in Table 3.

**Table 3:** Combined relative closeness of schemes.

Scheme	Positive Ideal Scheme Projection	Negative Ideal Scheme Projection	Combined relative proximity
I	0.0765	0.0981	0.4380
II	0.1096	0.1011	0.5202
III	0.0473	0.1037	0.3133

The comprehensive analysis reveals Scheme II as the optimal choice (relative closeness: 0.5202), followed by Schemes I and III. Scheme II prioritizes ecological preservation through underground tunneling within protected areas, significantly reducing environmental disruption while maintaining regional ecological balance. Geotechnical assessments confirm favorable tunnel conditions (Grade II-III surrounding rock) with minimized geological risks, avoiding steep slopes prone to rockfalls and debris flow accumulation zones. Despite extensive tunneling, the design achieves cost efficiency through minimized land acquisition and reduced demolition requirements, ensuring practical feasibility. The alignment of theoretical results with implemented engineering outcomes validates the decision model’s scientific rigor and practical applicability.

## 6. Conclusion

The study developed a 20-indicator evaluation framework for high-speed railway route planning in ecologically sensitive mountainous regions. Grounded in technical, economic, environmental, and social dimensions, this system prioritizes minimizing ecological disruption while addressing unique geographical challenges such as steep terrain and protected ecosystems.

A hybrid weighting system integrates subjective expertise (G2 method) and objective data patterns (CRITIC method). Relative entropy optimization resolves discrepancies between these approaches, ensuring scientifically robust weight allocation that accounts for both indicator variability and interdependencies.

The utility-based decision model evaluates alternatives through comprehensive relative closeness metrics. Validation via a case study demonstrated strong alignment between theoretical results and practical engineering outcomes, establishing a reliable framework for sustainable railway planning in challenging terrains.
